# The Modified Kimura's Technique for the Treatment of Duodenal Atresia

**DOI:** 10.1155/2009/175963

**Published:** 2009-05-17

**Authors:** Biagio Zuccarello, Antonella Spada, Antonio Centorrino, Nunzio Turiaco, Maria Rosaria Chirico, Saveria Parisi

**Affiliations:** ^1^Pediatric and Neonatal Surgery Unit, Department of Medical and Surgical Pediatrics, University of Messina, Policlinico Universitario G. Martino, Viale Gazzi 1, 98100 Messina, Italy; ^2^Department of Anesthesiology and Intensive Care, University of Messina, 98100 Messina, Italy

## Abstract

*Background/Purpose*. Kimura's diamond-shaped-duodenoduodenostomy (DSD) is a known technique for the correction of congenital intrinsic duodenal obstruction. We present a modification of the technique and review the advantages of this new technique. *Methods*. From 1992 to 2006, 14 newborns were treated for duodenal atresia. We inverted the direction of the duodenal incisions: a longitudinal incision was made in the proximal duodenum while the distal was opened by transverse incision. *Results*. Our “inverted-diamond-shaped-duodenoduodenostomy” (i-DSD) allowed postoperative oral feeding to start on days 2 to 3, peripheral intravenous fluids discontinuity on days 3 to 8 (median values 3.6); time to achieve full oral feeds on days 8 to 12 (median values 9.4); the length of hospitalisation ranged from 10 and 14 days (median value 11.2). No complications related to the anastomosis, by Viz leakage, dehiscence, biliary stasis, or stenosis were observed. *Conclusions*. The i-DSD provides a safe procedure to protect the ampulla of Vater from injury and avoids any formation of a blind loop. The results show that patients who have i-DSD achieve full oral feeds in a very short time period and, consequently, the length of hospitalisation is also significantly reduced.

## 1. Introduction

 For the surgical treatment of congenital intrinsic duodenal obstruction *KIMURA*, in 1977, introduced an anastomotic technique of side-to-side duodenoduodenostomy in two layers, arranging the bowel incisions to form a “diamond-shaped” (DSD) and created a larger stoma. In 1990, he refined his technique based on a transverse incision in the distal end of the proximal duodenum and a longitudinal incision in the distal duodenum. The double layer anastomosis was completed using 5–0 or 6–0 catgut or Vicryl continous inner and 6–0 silk interrupted outer layer sutures. No gastrostomy or transanastomotic tube was used. By this technique the anastomosis recovered its function in a significantly shorter time period and early postoperative feeding could be started. 

 In the same year, we adopted this new technique in 2 cases (in which we observed a start of alimentation after 3 and 4 days and postoperative duodenal-gastric reflux). 

In 1992, we modified the original Kimura's procedure in an inverted diamond-shaped duodenoduodenostomy (i-DSD). We present the technical points of the modification to the procedure and review the early advantages and the long-term bowel function in these patients.

## 2. Materials and Methods

### 2.1. Patients

From 1992 to 2006, 14 consecutives newborns (6 males and 8 females) were treated for total congenital intrinsic duodenal obstruction (Table 1). The mean gestational age was 38.1 weeks, the mean birth weight was 2715 g, and the mean age at operation was 1.75 days. All the patients presented *with* atresia of the second portion of the duodenum (DA). Maternal polydramnios was present in 9/14 (64.3%), and prenatal ultrasonography scan diagnosis of duodenal obstruction was available in 12/14 (85.7%). Eleven associated anomalies were found in 8 patients. Patients with associated anomalies which might affect oral feeding have been excluded from the survey, so that the number of variables that might affect the outcome are reduced to a minimum. (2 cases were excluded for imperforated anus, 1 Down's syndrome, and 1 severe congenital heart disease).

### 2.2. Surgical Technique

After proper preparation by nasogastric decompression and fluid and by electrolyte replacement the operation was carried out, under general endotracheal anesthesia, through a right transverse upper abdominal incision. The abdominal muscles were divided transversely with cutting diathermy and the peritoneal cavity was opened in the line of incision. The *hepatic* flexure of the colon was mobilized by reflecting it downwards to expose the dilated duodenum. The duodenum was then adequately mobilized by Kocher's manoeuvre. A soft rubber tube was inserted either by orogastric or gastrostomy and advanced into the duodenum to assess the level and nature of obstruction. The redundant wall of the proximal duodenum was brought down to overlie the proximal portion of the distal duodenal segment. If this could not be done easily, more megaduodenum was mobilised . The ligament of Treitz was divided in two cases, for more mobilization of the distal duodenum. 

**Figure 1:**
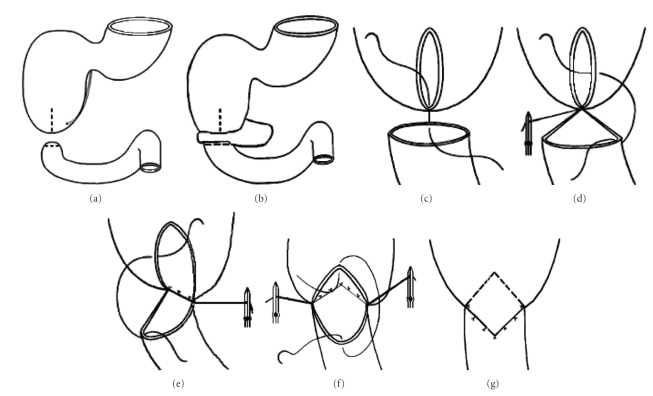
Personal modification (inverted diamond-shaped anastomosis): (a-b) longitudinal incision on the proximal dilated duodenum and transverse incision on the distal duodenum; (c-d-e-) anastomosis of posterior duodenal wall in a single layer with interrupted sutures; (f-g) anastomosis of the anterior duodenal wall.

 We modified the Kimura's procedure (Figure 1) by inverting the direction of the duodenal incisions. A *longitudinal incision *was made on the* proximal* dilated duodenum until the end of the blind pouch (or just close to annular pancreas, if present). After compression of the gallbladder, the papilla of Vater was localized by observing bile flow.The *distal* duodenum was opened by *transverse incision* at its top (or just close to annular pancreas). A mixture of air and saline was injected into the distal bowel lumen to rule out a distal obstruction. The distal duodenum was easily distended to a larger size during this manoeuvre by occluding the proximal jejunum and to withdrawing the *filled* (5 mL) Foley's balloon (Wangeesten's manoeuvre). The “inverted” anastomosis (i-DSD) was accomplished in a *single layer with interrupted 5–0 or 6–0 Vicryl sutures in an inverting fashion*. In* the *first 2 patients, we used 5–0 silk sutures. It started on the posterior duodenal wall by approximating the distal corner of the proximal longitudinal incision with the posterior midpoint of the distal tranverse incision. Then, each midpoint of the longitudinal incision was joined with the *corresponding* corner of the distal incision. The posterior wall was completed with intermediate *stitches.* At last, the anterior wall of the anastomosis was performed by approximating the uppermost corner of the longitudinal incision with the anterior midpoint of the distal incision and completed by intermediate stiches on each side. Neither duodenal tapering or transanastomotic tube or gastrostomy was used. Reconstruction of the abdominal wall was performed in layers using 4–0 Dexon sutures. The skin was closed with continous intradermic suture using 5–0 Dexon. 

In the immediate postoperative period the stomach was continuously emptied by gravity drainage via a nasogastric tube; when the gastric residual was less than 20 mL by passive drainage oral feeding was started with 30 mL of regular formula, which was progressiveley increased as tolerated, with concurrent scaling down of the intravenous feeding.

## 3. Results

In the present study 4 patients with associated anomalies (2 imperforated anus, 1 Down's syndrome, and 1 severe congenital heart disease) have been excluded from the survey. One patient died in the postoperative period due to associated cardiac anomaly. We analysed the most important parameters for the postoperative evaluation as day of starting of oral feeding, time to achieve full feeds, day of discontinuation of intravenous fluid, complications if any, and length of hospitalisation (Table 2).

 In the postoperative period the gastric residual usually stopped on day 1 to 2. All of the nine patients with i-DSD started oral feeding on days 2 to 3 (mean 2.1). The volume and concentration of the feeding were progressively increased, and full alimentation was achieved on days 8 to 12 (mean 9.4). On day 3 to 8, peripheral intravenous fluids were discontinued. We never used total parenteral nutrition (TPN). The patients did not show complications related to the duodenal anastomosis as leakage, dehiscence, spillage or stenosis, blind loop, and biliary stasis. The lenght of hospitalisation ranged from 10 to 14 days (mean 11.2).

 In the late follow-up a detailed history of morbidity and growth development were taken in addition to performance of clinical examination. All patients were followed in accordance to a protocol evaluating the esophageal function, the form of and the mucosal patterns of the stomach and duodenum, gastroesophageal and duodenogastric reflux, the model and speed of emptying of the stomach and the duodenum, by using x-ray series and ultrasonographic study, gastroesophageal pH-metry and duodenogastric manometry. The patients were free from gastrointestinal symptoms with growth development and body weight in normal range for age. Upper gastrointestinal contrast study showed passage of contrast material through the duodenal stoma. Duodenal diameter was found to show some decrease in size postoperatively and a trend towards normalisation over time. Abnormal morphology of the duodenum at the anastomosis persisted, without clinical discomfort, in 4 patients 4-5 aged years. In the oldest children this anatomical discrepancy progressively decreased to a size consistent with the age. 

In all patients ultrasound showed normal transit time. Gastroesophageal pH-metry showed absence of duodenogastric reflux and presence of gastroesophageal reflux in 1 case (12 months aged). The duodenal manometry did not show a reduced or absent contractile activity in the distal duodenum; in 2 patients we founded reduced contractile activity in the preanastomotic duodenal segment.

## 4. Discussion

Congenital intrinsic duodenal obstruction may be caused by duodenal atresia, stenosis, membrane, or web and most frequently occurs in the second part of the duodenum at or below the ampulla of Vater. 

 In the past, the transmesolic side-to-side duodenojejunostomy was the generally accepted procedure for the surgical treatment of the congenital intrinsic duodenal obstructions in the neonate [1]. Mortality and several anastomotic complications remained high until the introduction of the transanastomotic feeding tube and gastrostomy [2]. The results have markedly improved by better supportive management of high-risk neonates in the intensive care units, especially respiratory and nutritional support [3].

 The direct duodenoduodenostomy achieved good results [4, 5]. Neverthless, the literature's review highlighted complications related to the anastomosis. A stagnant pouch might predispose to the blind loop syndrome and persistant abnormal morphology of the duodenum in the late follow-up [6]. The deformity and dysfunction of the dilated duodenum were the causes of the morbidity and occasionally these patients required tapering [7] or duodenoplasty [8]. Others authors did not find any difference in patients undergoing either duodenoduodenostomy or duodenojejeunostomy in regard to lenght of time until onset of feeding, time to the discontinuation of intravenous feeds, or total hospitalisation time [9].

In 1977 Kimura [10] performed the diamond-shaped side-to-side duodenoduodenal anastomosis in nine consecutive cases of congenital duodenal obstruction and reported his experience over 15 years with 35 duodenal atresia using a diamond-shaped anastomosis. Arnbjörnsson [11] studied retrospectively 18 consecutives newborns with duodenal atresia, 9 from each of two different centres of pediatric surgery; Upadhyay [12] described 33 consecutives cases of duodenoduodenostomy (diamond-shaped anastomosis in 9 cases). Kimura's DSD reduced drastically the time of postoperative canalisation and achieved better results than previous types of duodenoduodenostomy**. **Barium studies in his series showed less deformed configuration of the duodenum [13]. The superiority of this “diamond-shaped anastomosis” was confirmed by Weber, but almost all his patients had a gastrostomy tube [14]. 

 Our technique is very similar to Kimura's DSD, except for the followed technically important changes.

1. The longitudinal incision on the proximal duodenum is very far from the outlet of the ampulla; it represents a safer procedure to protect the ampulla of Vater from injury; furthermore, the longitudinal incision until the end of the blind pouch removes any obstacle to duodenal transit and, thus, avoids formation of a blind loop. The same type of incision can be prolonged proximally in case of total or extramucosal plication duodenoplasty or prolonged on the distal duodenum and can represent the only step of the duodenotomy for duodenal web or membrane excision [15].
2. The transverse incision on the distal duodenum is sufficient for a large stoma because the manoeuvres *for inspection*, irrigation, and dilatation of distal bowel enlarge its size and stimulate postoperative bowel motility and early recovery of bowel function [4].
3. The single layer interrupted sutures anastomosis gives best blood circulation of the local tissues.

The greatest advantage is to avoid any obstacle (blind loop) to the intestinal transit and thus to achieve earlier recovery of anastomotic function and significantly less time to achieve full preanastomotic feeds (1-2 days) and shorter duration of hospital stay. All of the children have been followed to the present time, and so far none of them has experienced any problem related to our modified operative technique. The absence of anastomotic problems (dehiscence, stenosis, and biliary stasis) played a significant role to achieve the good result reported in this series. 

 Kimura found very low rate of complications and good long-term results [11]. In the experience of Kokkonen, although the great majority of his patients were symptom free, on barium meal examination all but two had abnormal findings and he concluded that some gastrointestinal disturbances are common even in asymptomatic patients, and careful follow-up is important [6]. Salonen reported the experience in a small group of 9 patients at age 3–21 years and founded in contrast a normal barium meal in all the groups except one [16].

 In our series abnormal duodenal morphology persisted in half of patients for 4-5 years; in the oldest children this discrepancy decreased progressively, suggesting that, in accordance with Kimura's experience, the DSD preserves a more natural anatomical configuration to the reconstructed duodenum. For this reason the tapering by excising a portion of the redundant wall of the proximal dilated duodenum increases the risk bowel spillage and damage the bile duct [17]. 

In conclusion, we believe that the “inverted diamond-shaped anastomosis” (i-DSD) can be applied to all types of intrinsic duodenal obstructions (i.e., atresia, stenosis, annular pancreas, duodenal web or membrane) and achieves very satisfactory result. The shorter time of hospitalisation also provides an evident benefit on the hospital cost.

## Figures and Tables

**Table 1:** Summary of clinical features in neonates with duodenal atresia (DA).

Total Patients:	
* *no.	14
Sex no. (%)	
* *male	6 (43.0)
* *female	8 (57.0)
Race no. (%)	
* *white	12 (85.0)
* *black	2 (15.0)
Polydramnios no. (%)	9 (64.3)
Gestational age-w	
* *mean (range)	38.1 (34–40)
Prenatal scan no. (%)	12 (85.7)
Birth weight-g	
* *mean (range)	2715 (2210–3630)
Associated anomalies no.	(11/in 8 pats)*

*4 patients were excluded: 2 imperforated anus, 1 Down's syndrome and 1 severe congenital heart disease.

**Table 2:** Procedures and results in patients operated on for DA.

Age at operation-day	
* *mean (range)	1.75 (1–3)
Type of technique (i-DSD) no.	14
Oral feeding start-day mean (range)	2.1 (2-3)
Intravenous fluid discontinuity-day	
* *mean (range)	3.6 (3–8)
Full oral feeds-day	
* *mean (range)	9.4 (8–12)
Length of hospitalization-day	
* *mean ( range)	11.2 (10–14)
Postoperative complications:	None
Late follow-up scan duodenal transit (time)	Normal
Abnormal duodenal morphology (<4-5 y)	
* *no. (%)	4 (44.5)
Duodenogastric reflux	none
Gastroesophageal reflux	
* *no. (%)	1 (11.1)
Reduced contractile activity	
* *no. (%)	2 (22.2)

## References

[1] Kraeger RR, Gromoljez P, Lewis JE (1973). Congenital duodenal atresia. *The American Journal of Surgery*.

[2] Becker JM, Schneider KM (1963). Tube jejunostomy in the treatment of upper intestinal obstruction in the neonate. *Surgery, Gynecology & Obstetrics*.

[3] Nerwich N, Shi E (1994). Neonatal duodenal obstruction: a review of 30 consecutive cases. *Pediatric Surgery International*.

[4] Weitzman JJ, Brennan LP (1974). An improved technique for the correction of congenital duodenal obstruction in the neonate. *Journal of Pediatric Surgery*.

[5] Girvan DP, Stephens CA (1974). Congenital intrinsic duodenal obstruction: a twenty year review of its surgical management and consequences. *Journal of Pediatric Surgery*.

[6] Kokkonen M-L, Kalima T, Jääskeläinen J, Louhimo I (1988). Duodenal atresia: late follow-up. *Journal of Pediatric Surgery*.

[7] Weisgerber G, Boureau M (1982). Resultats immediats et secondaires des duodeno-duodenostomies avec modelage dans le traitement des obstructions duodenales congenitales complete du nouveau-nè. *Chirurgie Pediatrique*.

[8] Dewan PA, Guiney EJ (1990). Duodenoplasty in the management of duodenal atresia. *Pediatric Surgery International*.

[9] Mooney D, Lewis JE, Connors RH, Weber TR (1987). Newborn duodenal atresia: an improving outlook. *The American Journal of Surgery*.

[10] Kimura K, Tsugawa C, Ogawa K, Matsumoto Y, Yamamoto T, Asada S (1977). Diamond-shaped anastomosis for congenital duodenal obstruction. *Archives of Surgery*.

[11] Arnbjörnsson E, Larsson M, Finkel Y, Karpe B (2002). Transanastomotic feeding tube after an operation for duodenal atresia. *European Journal of Pediatric Surgery*.

[12] Upadhyay V, Sakalkale R, Parashar K (1996). Duodenal atresia: a comparison of three modes of treatment. *European Journal of Pediatric Surgery*.

[13] Kimura K, Mukohara N, Nishijima E, Muraji T, Tsugawa C, Matsumoto Y (1990). Diamond-shaped anastomosis for duodenal atresia: an experience with 44 patients over 15 years. *Journal of Pediatric Surgery*.

[14] Weber TR, Lewis JE, Mooney D (1986). Duodenal atresia: a comparison of techniques of repair. *Journal of Pediatric Surgery*.

[15] Langer JC, Winthrop AL (1993). A simplified surgical technique for the management of gastrointestinal webs. *Pediatric Surgery International*.

[16] Salonen IS, Mäkinen E (1976). Intestinal blind pouch and blind loop syndrome in children operated previously for congenital duodenal obstruction. *Annales Chirurgiae et Gynaecologiae*.

[17] Ein SH, Shandling B (1986). The late nonfunctioning duodenal atresia repair. *Journal of Pediatric Surgery*.

